# Influence of the Die Height on the Density of the Briquette Produced from Shredded Logging Residues

**DOI:** 10.3390/ma14133698

**Published:** 2021-07-01

**Authors:** Tomasz Nurek, Arkadiusz Gendek, Magdalena Dąbrowska

**Affiliations:** Department of Biosystems Engineering, Institute of Mechanical Engineering, Warsaw University of Life Sciences–SGGW, Nowoursynowska 164, 02-787 Warsaw, Poland; tomasz_nurek@sggw.edu.pl (T.N.); magdalena_dabrowska@sggw.edu.pl (M.D.)

**Keywords:** die height, biomass compaction, relaxation coefficient, briquette density, logging residues, mechanical engineering

## Abstract

An alternative to plant biomass of various origins are forest logging residues. They differ significantly from other, previously used plant materials. This difference is due to the heterogeneous composition and relatively large size of individual particles. This research on the compaction of this type of shredded material was aimed at determining the influence of the die height on the density and relaxation of briquettes. This parameter is crucial for the proper construction of compaction devices. The measurements were carried out for the same fractional composition of the shredded logging residues, with variable input parameters of the material and process. It was found that the briquette density and relaxation are influenced by the die height, as well as the material moisture content and process temperature. The highest density at maximum compaction pressure (1.40 g·cm^–3^) was obtained at a moisture content of 16%, temperature of 80 °C, and the lowest die height (195 mm). In the case of the briquette density after ejection from the die, the best results were obtained at the same temperature and die height but at a moisture content of 9%. The tests confirmed that, regardless of the process temperature and material moisture, the briquette density increases as the die height is reduced. The relaxation coefficient of compacted logging residues ranges from 21.7% to 50.1% and depends mainly on the material moisture content and the temperature of the process. The lowest value of the relaxation coefficient (21.7 ± 1.61) was obtained at 9% moisture content, 60 °C temperature, and 220 mm die height.

## 1. Introduction

Logging residues are characterized by a heterogeneous composition. The content of wood, bark, and pine needles makes their chemical composition different from other traditional raw materials. Therefore, it becomes necessary to investigate and, consequently, develop new parameters for the compaction process of this type of biomass, as well as to develop design guidelines for compaction devices. Particular attention must be paid to the height of the compacting die, which affects the height of the biomass compacted using a piston in a closed die, or in the case of briquetting machines, in an open die.

For several years, energy policies pursued in the world have resulted in an increasing demand for plant biomass, which is intended for direct combustion or for processing into another type of fuel, including refined fuels. There are numerous scientific studies related to this topic that seek optimal process and material parameters, e.g., a reduction of processing costs, an increase in durability, and the energy obtained per unit volume of solid fuels in the form of briquettes or pellets.

Based on scientific reports, it can be concluded that tests of physical properties [1,2,3,4,5,6] (bulk density, moisture content, particle size), chemical properties [7,8,9,10,11,12,13,14,15,16,17,18] (carbon, hydrogen, nitrogen, ulphur oxygen and ash contents), and energy properties (gross and net calorific values) of plant biomass and other materials have been studied by multiple scientists.

The increasing demand for solid fuels in the form of briquettes and pellets is conducive to market development. In the case of the Polish market, there is a relatively large number of enterprises with a production of 1–5 thousand tons of briquettes or pellets per year, intended mainly for private use or for individual users on the local market [4]. Such enterprises have a low chance of purchasing sawdust for briquette production due to competition from large factories. Therefore, an opportunity for them to use other sources of raw material supply exists in shredded logging residues.

The by-products of the logging process are residues in the form of branches, treetops, and small trees and shrubs after the removal of undergrowth and saplings. This material, in its primary form or after shredding, can be used as a renewable energy source for direct combustion, or after processing, it can be used for the production of refined fuels in the form of briquettes [19]. Conversely, in the literature, there are multiple descriptions of research on the production of briquettes from plant materials of uniform composition and particle size [15,20,21]. The authors of the literature considered the parameters of the material and the process influencing the quality of the final product, such as moisture, particle size, temperature, pressure, and others.

Taulbee [20] indicates that the increase in process temperature causes an increase in the mechanical strength of briquettes. Higher temperatures cause plasticization of particles and activation of natural binders in the plant material [22]. Increased temperature during agglomeration is a desirable phenomenon. This causes hardening of the briquette, increasing its density, which is also confirmed by Kaliyan and Morey [23,24]. These researchers produced durable and dense briquettes from plant materials at a temperature of 65–100 °C. Similar conclusions were obtained by Wang et al. [25]; however, they conducted agglomeration at a temperature of 120 °C, while obtaining shorter compaction time.

An important factor that determines the mechanical properties of the briquette and its quality is particle size. Considering the use of shredded logging residues to briquette production, particle size depends on the type and construction of the shredder, the type of wood, the part of the tree that will be shredded, and the angle of setting and sharpening of the knife [7,26,27]. Barontini et al. [28] found that by shredding the wood with a blunt knife, smaller particles are obtained and the proportion of finer particles is increased. The fine particles during briquette production cause the briquettes to obtain higher density and durability [29,30].

During the agglomeration of plant materials, an important factor influencing the quality of the obtained briquette is moisture content. According to the conclusions presented by Kaliyan et al. [23,31], Wang et al. [25], and Gurdil and Melki [32], material moisture content should be within a range of 15–20%. An increase in moisture causes deterioration of the briquette quality [33,34] and adversely affects the compaction process [35]. Conversely, and based on their obtained results, Mani et al. [36] and Shaw et al. [37] stated that in order to obtain greater durability and density, agglomeration should be carried out at 5–10% moisture. However, Gendek et al. [38] produced briquettes from shredded cones with a moisture content of approx. 8%, achieving mechanical durability of briquettes at a level of 0–27%, and by increasing the moisture to approx. 15–20%, the briquette durability was above 87%.

The density of the final product depends on its relaxation. Wongsiriamuay and Tippayawong [39] found that a decrease in temperature causes an increase in relaxation of the pellets. For a temperature of 30 °C, relaxation was 16–30%, while at a temperature of 60–80 °C, relaxation did not exceed 5%. The influence of temperature on the size of briquette relaxation was also confirmed by Shaw and Tabil [37]. Kaliyan and Morey [29] obtained an average relaxation of briquettes composed of switchgrass within a range of 15–32%, which depended on particle size and material moisture. High moisture, large particles, and low agglomeration temperature results in a greater relaxation of the briquette.

The studies mentioned above were carried out mainly for the compaction of materials with a homogeneous composition, e.g., sawdust, crushed various parts of maize or energy crops, etc. Nevertheless, there is not enough research describing the possibilities of producing a briquette from shredded logging residues and the properties of the obtained briquette [19,40,41]. Authors indicated the optimal fractional composition of shredded logging residues, material moisture, and process temperature to obtain a briquette of appropriate density and durability. Depending on the assumed process parameters, they achieved a density ranging from approx. 800 kg·m^–3^ to approx. 1200 kg·m^–3^ and a durability coefficient of up to approx. 50–72%.

Taking into consideration the above information, the next stage of the authors’ research is to consider the changes in the height of the biomass bed by changing the die height to indicate how this height affects briquetting. Therefore, the aim of the research is to determine the effect of die height (biomass bed height) on the density of the produced briquette and its relaxation at a constant fractional composition, while considering the variable input parameters of the material and the briquetting process.

## 2. Materials and Methods

### 2.1. Research Stand

**Figure 1:**
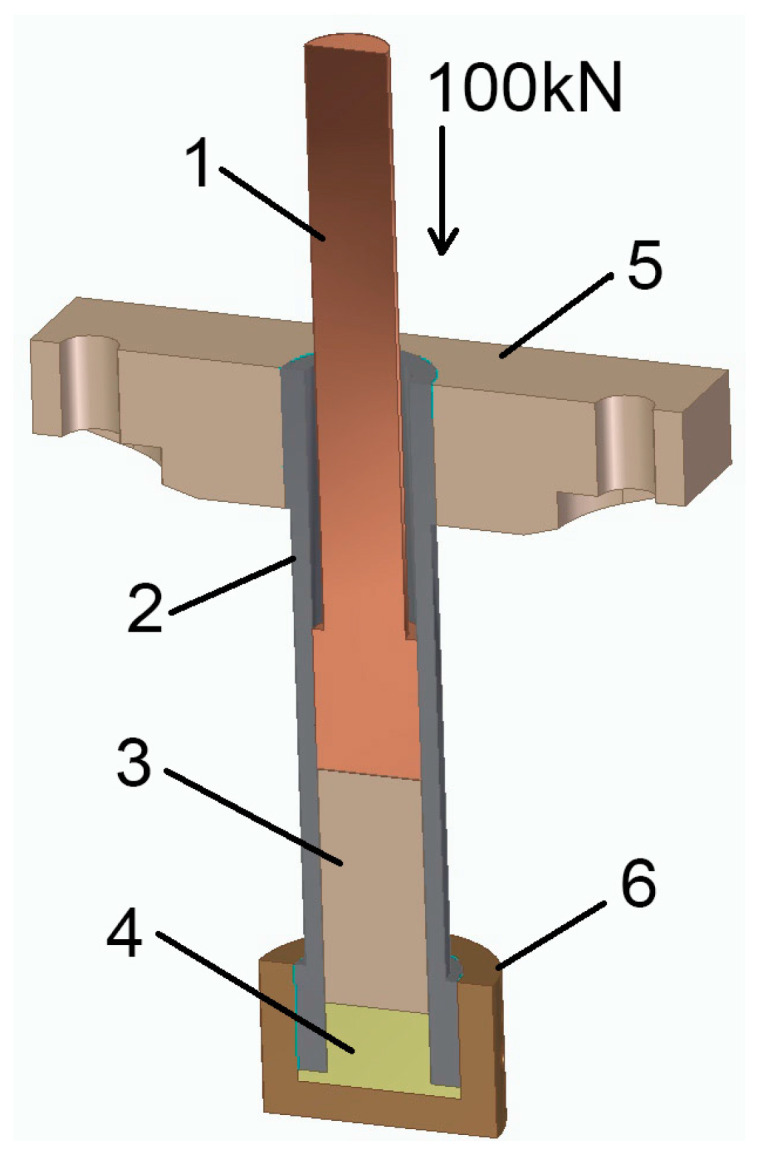
Construction and cross-section of the stand for biomass compaction. 1: piston; 2: bushing of compaction die; 3: compacted biomass; 4: replaceable spacer for adjustment of die height; 5: mounting for die; 6: die cover.

Biomass compaction was carried out in a closed die (Figure 1). A Veb Thüringer Industriewerk Rauenstein (TIRA GmbH, Schalkau, Germany) testing machine with a maximum pressure force of 100 kN was used for the tests. The stand was equipped with a measuring system, enabling the recording of results (HBM Catman v.2.1 program—Hottinger Baldwin Messtechnik GmbH, Darmstad, Germany). The temporary compaction force was recorded with a strain gauge placed on the piston with an accuracy of ±1 N, and the displacement of the piston was recorded using a displacement sensor with an accuracy of ±0.01 mm.

The parameters of the die enabled a maximum compaction pressure equal to p = 65 MPa. The total volume of the die was *V_k_* = 448.3 cm^3^. Its diameter was *d* = 45 mm and the maximum die height was *h* = 295 mm. The following values of the die height, identical to the bed height of the compacted biomass, were assumed: *h_z_* = 295, 270, 245, 220 and 195 mm. The effect of changing the bed height was achieved by filling the die with cylindrical spacers with heights of 25, 50, 75 and 100 mm composed of S235 steel. The speed of compaction was constant at 3.2 mm·s^−1^, and the compaction time, depending on the bed height, was within the range of 50–80 sec, while the total time of biomass remaining in the die was 3–4 min.

Two micanite band heaters with a total power of 600 W (ϕ60x25/230V300W, Selfa GE S.A., Bydgoszcz, Poland) were mounted on the outer surface of the die. The temperatures of heating the die set at *T* = 60, 80, 100, 120 °C were maintained with a range of ±1 °C by the EMKO ESM-3710 controller (EMKO Elektronik A.S., Bursa, Turkey).

### 2.2. Material

The investigated material was shredded logging residues. This biomass was collected directly from the forest area located in the Rajgród Forest District, Poland (GPS: N 53.4909, E 22.5825) in the form of branches left after harvesting the wood of *Pinus sylvestris* at the age of 86 years. It contained white wood, a considerable amount of pine needles, and small non-woody shoots. The collected branches and needles were shredded in a BT13HP-90 mm hammer shredder (Grupa REDMET sp.j., Dębica, Poland).

The particle size distribution of the shredded biomass was tested using the sieving method. The chips were divided into fractions using a sieve separator in accordance with ISO 17827-1: 2016 standard [42]. Screens produced in accordance with ISO 3310-2: 2013 [43] and ISO 565: 2000 standards [44] were used for separation. Detailed research methodology and construction of the stand were described by Lisowski et al. [45,46]. Based on the cited ISO 17827 standard, the value d_50_ = 5.86 mm was determined, i.e., the average size of the sieve through which 50% of the particles were passed.

### 2.3. Compaction Process

Compaction was carried out for two biomass moisture contents *MC* = 9% and 16%. Moisture content was controlled by the drying-weighing method according to the EN13183-1:2004 standard. Samples of 100 ± 0.5 g were placed in the laboratory drier UF55 plus (Memmert, Schwabach, Germany). The drying process at a temperature of 105 °C was carried out until the dry substance was obtained. The mass of samples and loss in moisture content was controlled with an accuracy of ±0.01 g using laboratory scales RADWAG WTC 600 (Radwag, Radom, Poland).

Moisture (*MC*, %) for shredded logging residues was determined using the formula:

(1) $$MC=\frac{m_{bw}-m_{bs}}{m_{bw}}\cdot 100,$$

where: $m_{bw}$ is a mass of wet sample (g), $m_{bs}$ is a mass of dry sample (g).

For two moistures, the bulk densities of tested chips were $\rho_{9}$ = 0.22 g·cm^–3^ and $\rho_{16}$ = 0.24 g·cm^–3^.

For individual measurement series (different die heights), to maintain the same initial conditions of compaction, i.e., the same density for different volumes, samples were prepared with the masses calculated from the following formula:

(2) $$m_{b}=\rho_{w}\cdot V_{k},$$

where: $m_{b}$ is a mass of tested sample (g), $\rho_{w}$ is a bulk density of wood chips for a specific moisture (g·cm^–3^), and $V_{k}$ is the total volume of compacting die (cm^3^).

After filling the compacting die at a predetermined temperature with a suitable mass of chips, the testing machine was started. The temporary value of the compaction force and the piston displacement were recorded automatically every 0.1 s until the maximum pressure P = 65 MPa was obtained. After stopping, the piston was retracted from the compacting die, and after opening the die, the briquette was pushed out of the bushing.

### 2.4. Determination of the Volume and Density of the Briquette

During the measurements, the briquette height was measured twice. The height at maximum compaction pressure (P = 65 MPa) *h*_0_ was measured when the briquette was inside the compaction die. This height was determined with an accuracy of 0.1 mm based on the reading from the apparatus used to measure piston displacement. The second measurement of the briquette height *h*_1_ was rendered using an electronic caliper with an accuracy of 0.5 mm immediately after the briquette was ejected from the die. Measurements were constructed in two perpendicular planes and the result was averaged.

Briquette volume (*V_i_*) and density ($\rho_{i}$) were determined based on the measurement of briquette height in the die (*h*_0_) and its height after rejection from the die (*h*_1_), and the mass of a single briquette ($m_{a}$) was determined every time by weighing the briquette using the formulas:

(3) $$V_{i}=\frac{\pi d^{2}h_{i}}{4}\;,\;$$

(4) $$\rho_{i}=\frac{m_{a}}{V_{i}}\;,\;$$

where: ‘*i*’ is the index (0 for briquette in the die, 1 for briquette ejected from the die), $V_{i}$ is the briquette volume (cm^3^), *d* is the die diameter (cm), and $h_{i}$ is the briquette height (cm).

Considering briquette density in the die ($\rho_{0}$) and after ejection from the die ($\rho_{1}$) the relaxation coefficient was determined, λ:

(5) $$\lambda =\frac{\rho_{1}-\rho_{0}}{\rho_{1}}\cdot 100\;,\;$$

For each of the combinations of input parameter values, a minimum of 10 repetitions was performed.

### 2.5. Statistical Analysis

The parameters were analyzed using the Statistica v.13 program (TIBCO Software Inc., Palo Alto, Santa Clara, CA, USA). Analyses of variances (ANOVA) were performed at a significance level of *p* = 0.05.

## 3. Results and Discussion

### 3.1. Briquette Density in the Die ρ_0_

**Figure 2:**
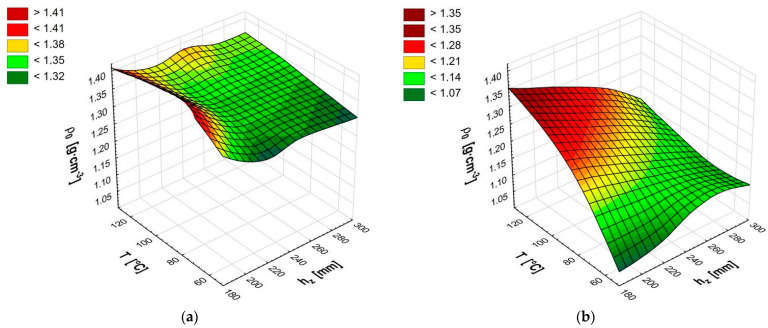
Briquette density inside the die at maximum pressure *ρ*_0_ for moisture content 16% (**a**) and moisture content 9% (**b**).

Briquettes obtained from a biomass with a higher moisture content (*MC* = 16%) were characterized by a slightly higher density than those obtained at a lower moisture content (Table 1). For the temperature T = 80 °C and the die height *h_z_* = 195 mm, density was *ρ*_0_ = 1.40 g·cm^–3^, while for the biomass with lower moisture *MC* = 9% it was on average *ρ*_0_ = 1.22 g·cm^–3^. The graph for the moisture content of *MC* = 16% (Figure 2a) is irregular, although it shares similarity with the graph for the moisture content *MC* = 9% (Figure 2b). In both cases, the density increased with increasing temperature, although for *MC* = 9%, it is more visible and consistent with previous studies by other authors [23,24,25,47]. For moisture *MC* = 16%, changes in the density values were not high. The maximum density of 1.40 g·cm^–3^ was obtained for the temperature T = 80 °C and the die height *h_z_* = 195 mm, and the lowest density *ρ*_0_ = 1.31 g·cm^–3^ was obtained for the temperature T = 60 °C and die height *h_z_* = 220 mm. The inclination of the graph plane showed a tendency of increasing briquette density composed of biomass with a moisture content *MC* = 16% with an increasing temperature. The effect of changes in the die height on the density was less visible than the effect of temperature.

Compacting the chips with a moisture content of 9% resulted in more regular changes in the briquette density (Figure 2b, Table 1). The maximum value of density *ρ*_0_ = 1.31 g·cm^–3^ was obtained for temperature T = 120 °C and die height *h_z_* = 195 mm. The density decreased with reduction in the temperature of the compaction process to T = 80 °C and an increase to the die height. The compaction of biomass at the lowest of the assumed temperatures (T = 60 °C) resulted in a significant reduction in density by about 15%, but negligible for a maximum die height *h_z_* = 295 mm of about 5%. This was related to the effect of “amortization” pressure in the upper layer of the biomass. The temperature T = 60 °C did not lead to full plasticization of resins and other substances contained in the biomass. The matter retained elastic properties, which resulted in the aforementioned effect of amortization and reduction of the degree of compaction.

The discussed briquette densities (1.2–1.4 g·cm^–3^) inside the die at a maximum pressure of approx. 63 MPa were similar to the values obtained by Gendek et al. [38] for briquettes from shredded spruce cones (1.1 g·cm^–3^). Similar densities (>1.0 g·cm^–3^) were obtained, among others, by Borowski [48] for briquettes composed from a mixture of coal with biomass, and Gürdil and Demirel [21] for briquettes composed from walnut shells. Such densities are characteristic for pellets composed from various plant materials [49,50,51], but in all cases the agglomerated particles were approx. 1 mm in size, and measurements were composed for briquettes and pellets outside the die.

Statistical analysis (ANOVA) showed the presence of the influence of all input parameters on the briquette density inside the die for the moisture content *MC* = 9%. In the case of moisture content *MC* = 16%, only for the combination of input parameters (bed height and temperature), was it indicated that they did not affect the tested parameter (*p* > 0.05), Table 2.

### 3.2. Briquette Density after Ejection from the Die ρ_1_

**Figure 3:**
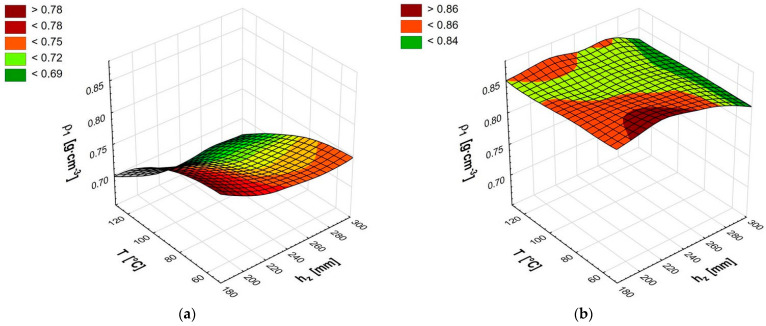
Briquette density after ejection from the die *ρ*_1_ for moisture content 16% (**a**) and moisture content 9% (**b**).

The briquette density after ejection from the die changed to a small extent. In the case of biomass moisture content *MC* = 16%, the lowest density *ρ*_1_ = 0.69 g·cm^–3^ was obtained for the temperature T = 120 °C in four out of five die heights. Only for the lowest die height *h_z_* = 195 mm was the density higher, only by 3%. The highest density *ρ*_1_ = 0.77 g·cm^–3^ was obtained for the temperature T = 60 °C and the small die height *h_z_* = 195 mm. Even less significant changes were observed for compaction of biomass with a moisture content *MC* = 9% (Figure 3, Table 1). On average, it was about 12% higher than the density for biomass with moisture *MC* = 16% and ranged from *ρ*_1_ = 0.83 to *ρ*_1_ = 0.87 g·cm^–3^. As shown in the graph for briquette density from biomass of moisture content *MC* = 9% (Figure 3b), there is no clear, unequivocal trend of changes. There is a weak tendency to decrease the density for the maximum die height. This confirms the results obtained in the study of the height of compacted biomass. The graph for the moisture content *MC* = 16% (Figure 3a) shows a lower briquette density obtained for higher temperatures. This is also confirmed by the results of the research on the die height (compacted biomass). Statistical analysis for both values of moisture (Table 3) showed the existence of the influence of temperature and die height on the briquette density (*p* < 0.05). An Analysis of the simultaneous influence of these parameters on the briquette density showed no such influence (*p* > 0.05).

The obtained density (0.7–0.87 g·cm^–3^) of the briquette immediately removed from the die was lower than the density of the briquette composed of shredded forest cones (0.94–1.1 g·cm^–3^) [38], and partially within the range or slightly below the density (0.8–1.0 g·cm^–3^) for briquettes composed of rice straw [25] and wood sawdust [32]. It is satisfactory that this density was higher than that of a briquette composed of tropical wood sawdust (0.62–0.72 g·cm^–3^) obtained by Mitchual et al. [52]. In every case, the authors compacted the shredded plant materials to particles of approx. 1 mm in size. However, referring to the ISO 17225 standard [53] describing the classification of briquettes, briquettes composed of shredded wood chips do not meet the requirements of this standard.

### 3.3. The Relaxation Coefficient λ

**Figure 4:**
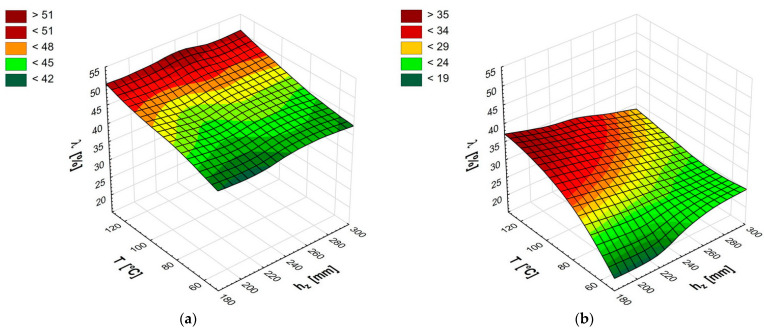
The changes of relaxation coefficient values [%] for moisture content 16% (**a**) and moisture content 9% (**b**).

By comparing of values of the relaxation coefficient presented in Figure 4 (Table 1), it can be concluded that in each of the considered cases, (combination of temperature and die height) a much more favorable situation (less relaxation) occurred for the moisture content *MC* = 9%. For moisture content *MC* = 16% (Figure 4a), the relaxation coefficient ranged from *λ* = 42.4% for the temperature T = 60 °C and the die height *h_z_* = 220 mm to *λ* = 50.1% for the temperature T = 120 °C and die height *h_z_* = 245 mm. Moreover, for moisture content *MC* = 9%, the minimum relaxation *λ* = 21.7% was obtained by the briquette produced at T = 60 °C and *h_z_* = 220 mm, and the maximum relaxation *λ* = 35.4% was obtained by the briquette produced at T = 120 °C and *h_z_* = 195 mm. For moisture content *MC* = 16%, a significant increase in the relaxation coefficient with increasing temperature was observed, which is the opposite of the phenomenon found by Kaliyan and Morey [29] during switchgrass briquetting and by Wongsiriamuay and Tippayawong [39] testing pellets composed of various parts of maize. However, these authors created briquettes and pellets from materials characterized by homogeneity and much smaller particle sizes. The increase in the value of the relaxation coefficient along with the increase in temperature had a negative effect, which may be linked with a strong, excessive liquefaction of the resins contained in the biomass and the resulting loss of the possibility of binding (sticking) the biomass immediately after leaving the compacting die [54]. Moreover, during compaction, an intense evaporation of water and other substances contained in the biomass occurred. After compaction and ejection, the briquette rapidly expanded by gases produced in the process of vaporization and its linear dimensions increased. Less significant changes in the relaxation coefficient occurred with a change in the die height. However, less relaxation was observed at a lower height for all tested temperatures. This change was around one percentage point. For moisture content *MC* = 16%, the statistical analysis showed that there was no influence of the die height and the interaction of temperature and the die height on the value of the relaxation coefficient. In both cases, the *p*-value was less than 0.05.

As noted for moisture content *MC* = 9% (Figure 4b), the relaxation coefficient was lower for all the values of the input parameters. In this case, the influence of the die height on the value of the relaxation coefficient was visible. In the case of high temperatures, reducing the die height had a negative effect (increasing the relaxation coefficient), while for lower temperatures, the effect was positive. The lowest value of the relaxation coefficient *λ* = 21.7% was obtained for the temperature T = 60 °C and the die height *h_z_* = 220 mm. An increase in temperature caused an increase in the value of the relaxation coefficient for each die height. This increase was clearer for the lower die height, from the value of *λ* = 22.5% for *h_z_* = 195 mm to *λ* = 35.4% for *h_z_* = 195 mm. For the die height *h_z_* = 295 mm, the relaxation coefficient varied from 24.5% to 28%.

The statistical analysis for moisture content *MC* = 9% confirmed the influence of all input parameters on the value of the relaxation coefficient (Table 4).

It can be assumed that for lower compaction temperatures up to T = 80 °C, the more advantageous design of compacting devices is at the lower die height. For high compaction temperatures less frequently used, the die height does not significantly affect the value of the relaxation coefficient.

## 4. Summary and Conclusions

Based on these studies, it can be concluded that shredded logging residues can be used to produce briquettes, but it is difficult to obtain briquettes of appropriate size and density over time. The density of the briquette obtained in the tests is lower than that required by the relevant standards ISO 17225-3 [53].

Studies have shown that better briquetting effects (higher density) are achieved at a lower temperature and for a small die height. In the case of moisture content *MC* = 16%, temperature T = 60 °C, and die height *h_z_* = 195 mm, the density was equal to *ρ*_1_ = 0.77 g·cm^−3^. However, for moisture content *MC* = 9%, the highest density *ρ*_1_ = 0.86 g·cm^−3^ was obtained for temperature T = 60 °C and die height *h_z_* = 220 mm. For die height *h_z_* = 195, the obtained density was only slightly lower (by 0.01 g·cm^−3^). In all test series, a decrease in density was observed with increasing die height. The obtained results are a direct indication for briquette manufacturers. It was found that for the type of tested biomass, it is beneficial to compact a portion of raw material. This design solution results in greater compaction and a lower degree of relaxation.

The research showed unequivocally that the compaction of biomass with a moisture content of 9% leads to briquettes of higher density, while increasing the temperature to 100–120 °C does not improve the compaction effects. An important result of the conducted research is the determination of the relaxation coefficient. The smallest change in density was observed for a moisture content of 9%, temperature of 60 °C, and die height of 220 mm. This indicates that increasing the process temperature has no benefit on improving the density or lowering energy consumption of the process. High temperatures may lead to a reduction in the bonding effect of the briquette, for example, causing scorched resin immediately after the briquette has been withdrawn from the die. Increased relaxation at high temperatures may also be the result of rapid evaporation of water and other substances contained in the compacted biomass.

## Figures and Tables

**Table 1:** The results of changes in briquette densities.

T, °C	*hz*, mm	*ρ*_0_, g·cm^–3^					*ρ*_1_, g·cm^–3^					*λ*, %				
		Mean	SD	−95%	+95%	SE	Mean	SD	−95%	+95%	SE	Mean	SD	−95%	+95%	SE
*MC* = 16%				-												
60	195	1.36	0.07	1.29	1.43	0.03	0.77	0.02	0.75	0.79	0.01	42.8	3.63	39.03	46.64	1.48
	220	1.31	0.04	1.27	1.36	0.02	0.75	0.04	0.72	0.79	0.01	42.4	4.06	38.18	46.69	1.66
	245	1.33	0.04	1.29	1.37	0.02	0.75	0.02	0.72	0.77	0.01	43.9	2.74	41.04	46.79	1.12
	270	1.32	0.02	1.30	1.34	0.01	0.74	0.02	0.72	0.76	0.01	43.9	2.17	41.62	46.18	0.89
	295	1.31	0.03	1.28	1.35	0.01	0.74	0.02	0.73	0.76	0.01	43.2	2.73	40.32	46.04	1.11
80	195	1.40	0.04	1.36	1.45	0.02	0.76	0.01	0.75	0.78	0.00	45.6	1.96	43.58	47.69	0.80
	220	1.34	0.03	1.31	1.37	0.01	0.75	0.01	0.74	0.75	0.00	44.3	1.27	43.00	45.66	0.52
	245	1.34	0.02	1.32	1.36	0.01	0.72	0.03	0.70	0.75	0.01	45.8	2.33	43.31	48.19	0.95
	270	1.34	0.02	1.32	1.35	0.01	0.73	0.03	0.70	0.76	0.01	45.5	2.56	42.76	48.14	1.05
	295	1.33	0.03	1.29	1.36	0.01	0.74	0.02	0.72	0.75	0.01	44.5	2.28	42.06	46.84	0.93
100	195	1.39	0.05	1.34	1.44	0.02	0.74	0.02	0.72	0.76	0.01	46.5	1.02	45.46	47.60	0.42
	220	1.33	0.02	1.31	1.35	0.01	0.73	0.02	0.71	0.75	0.01	45.0	1.18	43.72	46.21	0.48
	245	1.34	0.07	1.34	1.42	0.03	0.72	0.02	0.70	0.73	0.01	46.7	3.33	43.17	50.16	1.36
	270	1.34	0.04	1.32	1.38	0.02	0.72	0.02	0.70	0.74	0.01	46.3	2.84	43.36	49.31	1.16
	295	1.34	0.03	1.34	1.37	0.01	0.71	0.03	0.69	0.74	0.01	46.9	1.94	44.87	48.93	0.79
120	195	1.39	0.04	1.34	1.43	0.02	0.71	0.03	0.68	0.74	0.01	48.9	1.30	47.50	50.23	0.53
	220	1.35	0.04	1.31	1.39	0.02	0.69	0.01	0.68	0.70	0.00	48.8	2.02	46.69	50.94	0.83
	245	1.38	0.04	1.34	1.42	0.02	0.69	0.02	0.67	0.71	0.01	50.1	1.49	48.55	51.68	0.61
	270	1.35	0.03	1.32	1.38	0.01	0.69	0.03	0.67	0.72	0.01	48.6	2.9	45.52	51.61	1.18
	295	1.35	0.02	1.34	1.37	0.01	0.69	0.02	0.67	0.71	0.01	49.2	1.80	47.32	51.08	0.73
*MC* = 9%																
60	195	1.11	0.01	1.10	1.12	0.00	0.86	0.01	0.85	0.87	0.00	22.5	1.23	21.70	23.27	0.36
	220	1.11	0.01	1.10	1.12	0.00	0.87	0.02	0.86	0.88	0.01	21.7	1.61	20.62	22.68	0.47
	245	1.13	0.02	1.12	1.14	0.01	0.86	0.01	0.85	0.86	0.00	24.2	1.50	23.26	25.17	0.43
	270	1.13	0.02	1.12	1.14	1.01	0.85	0.01	0.84	0.86	0.00	24.8	1.25	23.97	25.55	0.36
	295	1.10	0.02	1.09	1.11	0.01	0.83	0.01	0.83	0.84	0.00	24.5	1.41	23.65	25.44	0.41
80	195	1.22	0.02	1.20	1.23	0.01	0.86	0.02	0.84	0.87	0.01	29.6	2.05	28.25	30.86	0.59
	220	1.19	0.03	1.17	1.21	0.01	0.86	0.02	0.85	0.87	0.00	28.0	2.38	26.50	29.53	0.69
	245	1.15	0.03	1.13	1.17	0.01	0.85	0.02	0.84	0.96	0.00	26.4	2.21	24.94	27.76	0.64
	270	1.12	0.01	1.12	1.13	0.00	0.85	0.01	0.84	0.86	0.00	24.3	1.61	23.30	25.34	0.46
	295	1.10	0.01	1.10	1.11	0.00	0.83	0.01	0.83	0.84	0.00	24.4	1.12	23.71	25.14	0.32
100	195	1.27	0.05	1.24	1.30	0.01	0.85	0.01	0.84	0.86	0.00	33.1	2.53	31.49	34.71	0.73
	220	1.23	0.05	1.21	1.26	0.01	0.84	0.02	0.83	0.85	0.00	31.9	3.45	29.70	34.08	1.00
	245	1.21	0.02	1.20	1.22	0.01	0.85	0.02	0.84	0.86	0.00	29.9	1.53	28.88	30.82	0.44
	270	1.18	0.02	1.16	1.19	0.01	0.84	0.01	0.83	0.85	0.00	28.5	1.56	27.55	29.53	0.45
	295	1.13	0.02	1.12	1.14	0.01	0.83	0.01	0.83	0.84	0.00	26.3	1.44	25.35	27.18	0.42
120	195	1.31	0.02	1.30	1.33	0.01	0.85	0.02	0.84	0.86	0.00	35.4	1.90	34.14	36.56	0.55
	220	1.28	0.02	1.27	1.30	0.01	0.85	0.02	0.84	0.86	0.00	33.4	1.61	32.41	34.46	0.47
	245	1.25	0.01	1.24	1.26	0.00	0.84	0.02	0.83	0.86	0.01	32.4	1.81	31.28	33.81	0.52
	270	1.21	0.02	1.20	1.22	0.00	0.85	0.01	0.84	0.86	0.00	29.8	1.52	28.87	30.81	0.44
	295	1.16	0.01	1.15	1.17	0.00	0.83	0.01	0.82	0.84	0.00	28.0	1.61	27.01	29.06	0.47

Note: SD: standard deviation; +95% and −95%: confidence interval; SE: standard error.

**Table 2:** Results of ANOVA analysis for density of briquettes in the die *ρ*_0_ at two moisture contents.

	df	SS	MS	F	*p*-Value
*MC* = 16%					
Die height (A)	4	0.0457	0.0114	7.9	0.000
Temperature (B)	3	0.0215	0.0072	5.0	0.003
Interaction (A × B)	12	0.0085	0.0007	0.5	0.915
*MC* = 9%					
Die height (A)	4	0.3069	0.0767	150.9	0.000
Temperature (B)	3	0.5617	0.1872	368.2	0.000
Interaction (A × B)	12	0.1276	0.0106	20.9	0.000

Note: df: degrees of freedom; SS: sum of squares, MS: mean square, F: F test value; *p*-value: significance level.

**Table 3:** Results of ANOVA analysis for density of briquette after ejection from the die *ρ*_1_ at two moisture contents.

	**df**	**SS**	**MS**	**F**	***p*-Value**
*MC* = 16%					
Die height (A)	4	0.0145	0.0036	8.0	0.000
Temperature (B)	3	0.0567	0.0189	41.6	0.000
Interaction (A × B)	12	0.0016	0.0001	0.3	0.991
*MC* = 9%					
Die height (A)	4	0.0142	0.0036	17.4	0.000
Temperature (B)	3	0.0032	0.0011	5.2	0.002
Interaction (A × B)	12	0.0043	0.004	1.7	0.061

**Table 4:** Results of ANOVA analysis for the relaxation coefficient for two moistures.

	df	SS	MS	F	*p*-Value
*MC* = 16%					
Die height (A)	4	26.7	6.7	1.14	0.341
Temperature (B)	3	542.1	180.7	30.93	0.000
Interaction (A × B)	12	17.3	1.4	0.25	0.995
*MC* = 9%					
Die height (A)	4	5355	1339	39.32	0.000
Temperature (B)	3	24,249	808.3	237.41	0.000
Interaction (A × B)	12	5603	46.7	13.71	0.000

## Data Availability

Not applicable.
